# A novel CD112-derived peptide targeting gut-primed neutrophils to attenuate deadly hepatic injury

**DOI:** 10.1186/s10020-026-01426-3

**Published:** 2026-02-17

**Authors:** Takayuki Kato, Atsushi Murao, Alok Jha, Gaifeng Ma, Monowar Aziz, Ping Wang

**Affiliations:** 1 Center for Immunology and Inflammation, The Feinstein Institutes for Medical Research, 350 Community Dr, Manhasset, New York 11030 USA; 2 Departments of Surgery and Molecular Medicine, Zucker School of Medicine at Hofstra/Northwell, Manhasset, New York USA

**Keywords:** Gut-liver crosstalk, Neutrophil, IEL, Liver injury, NETs, CD112

## Abstract

**Background:**

Gut-liver crosstalk exacerbates hepatic injury in gut ischemia/reperfusion (I/R), but its mechanism and therapeutic intervention remain elusive. Given the important roles of neutrophils and gut-resident intraepithelial lymphocytes (IELs) during acute inflammation, we hypothesized that the interaction between these two cell types worsens liver injury during gut I/R.

**Methods:**

Gut I/R was induced in mice by occluding superior mesenteric artery (SMA) for 60 min, followed by 4 h resuscitation. Blood, intestine and liver tissues were collected for various analysis. Neutrophil extracellular traps (NETs) were determined by microscopy. Gut I/R mice were injected (i.p.) with DPX2 (1 µg/g BW) at the time of reperfusion. After 4 h, blood and liver tissues were collected for various analysis.

**Results:**

We discovered that neutrophils in contact with IELs in gut I/R mice had increased ability to form NETs. As such, NETs^+^ neutrophils were increased in the portal vein blood of gut I/R mice compared to sham mice and the systemic blood, accompanied by increased NETs in the liver, indicating the migration of the activated neutrophils from the gut to the liver. Adoptive transfer of IEL-primed neutrophils into gut I/R mice exacerbated liver inflammation as indicated by increased liver tissue levels of inducible nitric oxide synthase (iNOS), interleukin-6 (IL-6), interleukin-1 beta (IL-1β), keratinocyte-derived chemokine (KC), and C-X-C motif chemokine ligand 2 (CXCL2). The interaction of neutrophils and IELs is known to be mediated via neutrophil CD112. Increased numbers of CD112^+^ neutrophils were observed in the gut epithelium after gut I/R. We have discovered a CD112-derived peptide, named DPX2, which inhibits the interaction between CD112 on neutrophils and its proinflammatory ligand CD226 on IELs. In vitro, DPX2 attenuated IEL-induced NETosis under inflammatory conditions. In vivo, the administration of DPX2 significantly decreased NET-forming neutrophils in the portal vein of gut I/R mice. In parallel to NETs inhibition, DPX2 administration significantly mitigated the gene expression of iNOS, IL-6, IL-1β, KC, and CXCL2 in the liver. Furthermore, the administration of DPX2 significantly attenuated liver injury as indicated by decreased serum aspartate aminotransferase (AST) and alanine aminotransferase (ALT), tissue injury score, and liver cell death.

**Conclusions:**

Neutrophil-gut IEL interaction mediates proinflammatory gut-liver crosstalk and the novel CD112-derived peptide DPX2 targeting this interaction has the potential to mitigate hepatic injury.

**Supplementary Information:**

The online version contains supplementary material available at 10.1186/s10020-026-01426-3.

## Introduction

Liver injury that develops secondary to systemic inflammation critically influences patient outcomes (Horvatits et al. 2019; Perez Ruiz et al. 2022). The liver plays a pivotal role in maintaining homeostasis by synthesizing essential proteins, metabolizing nutrients, and detoxifying harmful substances. Accordingly, liver dysfunction is a key determinant of disease severity and is included as a major component of scoring systems that quantify the severity of organ dysfunction or failure in critically ill patients, such as the Sequential Organ Failure Assessment (SOFA) score (Singer et al. 2016). Even in the absence of direct hepatic insults, inflammatory responses originating from extrahepatic organs, particularly the gut, are known to trigger secondary liver dysfunction—a phenomenon referred to as the gut–liver axis or gut–liver crosstalk (Tilg et al. 2022; Albillos et al. 2020) since gut and liver are anatomically and functionally connected via the portal circulation.

Neutrophils are key effector cells in acute inflammation (Kolaczkowska and Kubes 2013; Amulic et al. 2012; Clark et al. 2007). In addition to causing tissue damage through phagocytosis, degranulation, and NETs release, some neutrophils that escape out of the primary tissue re-enter the circulation (Woodfin et al. 2011), spreading inflammation to other organs, and affecting immune cells. NETs are large extracellular meshworks composed of cytoplasmic and granular proteins that neutralize and kill foreign substances, preventing the spread of bacteria and fungi (Papayannopoulos 2018). While they function as antibacterial defenses, excessive NET formation has been implicated in tissue damage and organ failure during systemic inflammatory responses (Clark et al. 2007; Yipp and Kubes 2013), such as ischemia-reperfusion and sepsis, and their control may be therapeutically useful (Papayannopoulos 2018). Among neutrophil surface molecules, CD112 (nectin-2) has emerged as an important immune checkpoint ligand that interacts with CD226 expressed on immune cells, such as lymphocytes (Murata et al. 2023; Zhu et al. 2016). CD112^+^ neutrophils have been reported to promote inflammatory responses during systemic infections, suggesting them as novel therapeutic targets (Murata et al. 2023).

Intraepithelial lymphocytes (IELs) are specialized T cells that reside within the intestinal epithelial layer (Cheroutre et al. 2011; Vandereyken et al. 2020). The human intestinal mucosa has an extensive surface area of approximately 200–400 square meters, composed of a single layer of epithelial cells that line the intestinal lumen (Cheroutre et al. 2011). Under normal physiological conditions, this epithelial layer is constantly exposed, from birth to death, to commensal microorganisms, dietary antigens, and environmental factors. Its primary function is to form a physical barrier between the body’s internal environment and the outside world, serving as the largest entry point for potential pathogens (Vandereyken et al. 2020; Ma et al. 2021; Sujino et al. 2016; Hoytema et al. 2017). It is estimated that roughly one IEL is present for every ten epithelial cells in the small intestine, and IELs comprise one of the most abundant T cell populations in the body (Ma et al. 2021; Hoytema et al. 2017; Kaer and Olivares-Villagómez 2018).

Due to their abundance and unique localization, IELs represent a distinct immune cell population that, although expressing activation markers, remain in a quiescent state—hence described as “activated yet resting” (Cheroutre et al. 2011; Hoytema et al. 2017). They play critical roles in both intestinal immune responses and the maintenance of mucosal immune homeostasis. Upon activation, IELs release inflammatory mediators and engage in direct cell-to-cell interactions with other immune cells. Recently, intestinal IELs have been found to interact with neutrophils to induce NETosis through neutrophil CD112 in sepsis (Murao et al. 2025).

In this study, we hypothesized that neutrophils interact with IELs in the intestinal mucosa, leading to the formation of NETs that subsequently propagate inflammation to the liver via the portal circulation. We further explored the contribution of CD112-mediated neutrophil–IEL interactions to this process and evaluated the therapeutic potential of disrupting CD112–CD226 signaling using a novel CD112-derived peptide, DPX2. This study provides novel insights into how intestinal immune–neutrophil crosstalk amplifies systemic inflammation and identifies neutrophil CD112 as a potential target to mitigate gut–liver inflammatory injury.

## Materials and methods

### Experimental animals

C57BL/6 mice (8–12 weeks old, weighing 20–28 g) were purchased from Charles River Laboratories (Wilmington, MA) and housed under standard conditions with a 12-hour light/dark cycle, with free access to water and standard mouse chow. A minimum of 3 days of quarantine and acclimatization was performed before experiments. Because sex differences influence the severity of gut ischemia/reperfusion (I/R) injury in both humans and mice, only male mice were used in this study to eliminate sex as a source of variability (Hundscheid et al. 2020; Wu et al. 2021).

### Ethics declaration

All experiments were approved by the Institutional Animal Care and Use Committee (IACUC) at the Feinstein Institutes for Medical Research (Protocol number: 24–0620).

### Gut ischemia-reperfusion (gut I/R) model

The gut ischemia-reperfusion model was established as previously described (Denning et al. 2020; Kobritz et al. 2022). Briefly, mice were anesthetized with 2–4% isoflurane inhalation, and spontaneous respiration was monitored throughout the surgery. After confirming anesthesia, a midline laparotomy was performed, and the superior mesenteric artery (SMA) was occluded with a non-traumatic vascular clamp (Cat. No.: 18055-04; Fine Science Tools, Foster City, CA). Ischemia was maintained for 60 min, after which the clamp was removed to establish reperfusion. CD112 decoy peptide DPX2 (1.0 µg/g; GenScript Biotech, Piscataway, NJ; lot number: U2759UGBG0-3/PE8562; HPLC purity: 96.4%), dissolved in PBS (0.2 mg/mL), or vehicle was administered intraperitoneally. DPX2 or vehicle (PBS) was administered immediately after the establishment of reperfusion, and the wound was closed. For resuscitation and analgesia, 1.0 mL of normal saline and 0.05 µg/g of buprenorphine were administered via subcutaneous injection, respectively. After surgery, mice were transferred to recovery cages with free access to food and water. Body temperature was maintained at 37 °C using a circulating warm water pad during surgery and for 1–2 h post-surgery. In the sham group, anesthesia and laparotomy were performed, but the SMA was not occluded. Four hours after reperfusion, mice were euthanized, and blood, intestines, and liver were collected.

### Isolation of intraepithelial lymphocytes (IELs)

IELs from the whole small intestine were isolated following a modified version of the methods described before (Mucida et al. 2013; Moon et al. 2021). Briefly, after carefully removing fat and Peyer’s patches, the whole small intestines were opened longitudinally, and luminal contents were gently flushed out with cold PBS. The ileal segments were then incubated at room temperature for 20 min in RPMI 1640 medium supplemented with 2% fetal bovine serum (FBS), 5 mM EDTA (Thermo Fisher Scientific, MA), and 1 mM DTT (Thermo Fisher Scientific) to detach the epithelial layer. The resulting tissue fragments were filtered through a 100 μm mesh to remove debris. IELs were subsequently purified by density gradient centrifugation using Percoll (Cytiva, MA). Specifically, cells suspended in 40% Percoll were overlaid with a 75% Percoll solution and centrifuged at 800 × g for 20 min at 20 °C. The interphase containing IELs was collected and washed for further analysis.

### Evaluation of neutrophil extracellular traps (NETs) in peripheral blood and liver

Blood samples were collected from the portal vein or heart 4 h after reperfusion in the gut I/R group, or at the corresponding time point in the sham group. Neutrophils were isolated using the EasySep™ Mouse Neutrophil Enrichment Kit (STEMCELL Technologies, Vancouver, BC, Canada) according to the manufacturer’s instructions. Isolated neutrophils were stimulated with 100 nM phorbol 12-myristate 13-acetate (PMA) for 12 h to induce NET formation. Subsequently, cultured neutrophils were incubated with 100 nM SYTOX Green (Cat. No.: S7020; Thermo Fisher Scientific, Waltham, MA, USA) for 30 min and examined under a fluorescence microscope (EVOS FL Auto Imaging System, Thermo Fisher Scientific). NETs in the liver were also determined and the proportion and absolute count of NETs⁺ cells were then compared between the sham and gut I/R groups.

### Neutrophil and tissue staining

Cultured neutrophils were incubated with 100 nM SYTOX Green for 30 min and observed using an EVOS FL Auto Imaging System fluorescence microscope (Thermo Fisher Scientific). Gastrointestinal tissue sections were stained with an APC anti-Ly6G antibody (Cat. No.: 127614, BioLegend, San Diego, CA) and DAPI (4’,6-diamidino-2-phenylindole), and observed using an LSM900 confocal microscope (ZEISS, Oberkochen, Germany). Single-cell suspensions of the gut epithelium of sham and gut I/R mice were stained with APC anti-Ly6G and BV 421 anti-CD112 (Cat. No.: 748046; BD Biosciences, San Jose, CA) antibodies to count neutrophils. Acquisition was performed using a BD LSR Fortessa (BD Biosciences), and data were analyzed by FlowJo software (Tree Star, Ashland, OR). Cell numbers were calculated by using Precision Count Beads (Cat. No.: 424902; BioLegend).

### Coculture of neutrophils and IELs

Bone marrow-derived neutrophils (BMDNs) were cocultured with or without intraepithelial lymphocytes (IELs) at 1:1 ratio and treated with 10 µg/mL of DPX2 or a scrambled peptide (SEGRPDSDVYPYI; GenScript Biotech) in the presence of 100 ng/mL LPS (MilliporeSigma) or under hypoxia-reoxygenation. After 12 h of coculture, NETs were evaluated by microscopy and neutrophils were sorted by fluorescence-activated cell sorting (FACS) using a BD FACSAria™ (BD Biosciences).

### Fluorescence-activated cell sorting

Cells were then blocked with Fc Receptor Block (clone: S17011E; Cat.No.: 156604; 1:200; BioLegend) and stained with Ly6G (clone: 1A8; APC; Cat. No.: 127614; 1:100; BioLegend) and LIVE/DEAD™ Fixable Violet Dead Cell Stain Kit (L34964, Thermo Fisher Scientific). The representative gating strategy is shown in Supplementary Fig. 1. A BD FACSAria™ III cell sorter was utilized to yield the required neutrophils for adoptive transfer studies (10^6^ cells per mouse), with post-sort purity consistently exceeding 99%.

### Adoptive transfer of neutrophils into gut I/R mice

WT mice were subjected to gut I/R and injected intraperitoneally with 10^6^ neutrophils cultured with or without IELs in the presence of LPS, and FACS-sorted following the gating strategy in Supplementary Fig. 1. Post-sort purity consistently exceeded 99%, and viability was above 95% before injection as assessed by trypan blue staining. 20 h after the surgery, the liver was harvested to evaluate the mRNA expression.

### RNA isolation and real-time quantitative PCR

Total RNA was extracted from homogenized liver tissues using TRIzol reagent (Invitrogen, Thermo Fisher Scientific). cDNA was synthesized using murine leukemia virus transcriptase (Biosystems, Thermo Fisher Scientific), and PCR was performed with forward and reverse primers and SYBR Green PCR master mix (Biosystems) using a StepOne-Plus real-time PCR machine (Biosystems). Mouse β-actin served as an endogenous control to normalize mRNA levels using the comparative Ct method. The sequences of primers are as follows: β-actin, (forward) CGTGAAAAGATGACCCAGATCA, (reverse) TGGTACGACCAGAGGCATACAG; iNOS, (forward) GGCAAACCCAAGGTCTACGTT, (reverse) GAGCACGCTGAGTACCTCATTG; IL-6, (forward) CCGGAGAGGAGACTTCACAG, (reverse) GGAAATTGGGGTAGGAAGGA; IL-1β, (forward) CAGGATGAGGACATGAGCACC, (reverse) CTCTGCAGACTCAAACTCCAC; KC, (forward) GCTGGGATTCACCTCAAGAA, (reverse) ACAGGTGCCATCAGAGCAGT; CXCL2, (forward) CCAACCACCAGGCTACAGG, (reverse) GCGTCACACTCAAGCTCTG.

### Computational modeling and synthesis of DPX2

The amino acid sequences of CD112 (P32507) and CD226 (Q8K4F0) were retrieved from the UniProt database. The structural models of CD112 and CD226 were generated by using Iterative Threading ASSEmbly Refinement (I-TASSER) (Yang et al. 2015). The model structures were constructed using templates with maximum percentage identity, sequence coverage, and confidence. The models were refined based on repetitive relaxations by short molecular dynamics simulations for mild (0.6 ps) and aggressive (0.8 ps) relaxations with a 4-fs time step after structure perturbations. The refinement of the model resulted in the enhancement of certain parameters, including Rama-favored residues and a decrease in poor rotamers. The CD112-CD226 complex was generated by using the ATTRACT and InterEvDock tools (Schindler et al. 2015; Quignot et al. 2021). The interaction between CD112 and CD226 was calculated using the PDBePISA tool, and the complex structure was visualized using PyMOL (Krissinel and Henrick 2007). Based on these analyses, DPX2 was developed as a CD112-derived peptide. Specifically, DPX2 was designed as a peptide corresponding to the sequence 249-RYPPEVSISGYDD-261 present on CD112, which is identified as the binding region for CD226.

### Surface plasmon resonance

Surface plasmon resonance (SPR) (OpenSPR, Nicoya, ON, Canada) was used to determine DPX2’s effect on the interaction between CD112 and CD226. rmCD112 (Cat. No.: 3869-N2-050) and CD226 (Cat. No.: 4436-DN-050) were purchased from R&D Systems (Minneapolis, MN). rmCD112 was immobilized to channel 2 and rmCD226 preincubated with or without DPX2 2.5 μM were injected as an analyte. Binding reactions were carried out at a flow rate of 30 uL/minute at 20 °C. The channel-1 was used as a control to evaluate nonspecific binding. The real-time interaction data were analyzed by TraceDrawer (Nicoya). The signals from the channel-1 were subtracted from the channel-2. Data were globally fitted for 1:1 binding (one-to-one model).

### Aspartate aminotransferase and Alanine aminotransferase

Colorimetric measurements of aspartate aminotransferase (AST) and alanine aminotransferase (ALT) were performed on plasma collected 4 h after reperfusion. At the time of sacrifice, whole blood was collected by cardiac puncture into a heparinised syringe and subjected to centrifugation at 1000 g for 15 min at 4 °C. The resulting plasma was stored at a temperature of -80 °C. The AST (Ref. No.: A7561-450) and ALT (Ref. No.: A7526-450) reagents were sourced from Pointe Scientific, Canton, MI. Assays were performed in accordance with the manufacturer’s protocol.

### Histological assessment of liver tissue injury

Liver tissue was harvested after 4 h of reperfusion and immediately preserved in 10% formalin before being embedded in paraffin. Biopsy tissue was sectioned at 5 µm and stained with hematoxylin and eosin (H&E) by AML Laboratories (Augustine, FL). Liver parenchymal injury was assessed in a blinded manner using semiquantitative light microscopy. The histological damage score for each sample was calculated based on the Suzuki scoring system, which is a scoring system for liver injury during hepatic I/R. The score was expressed as the sum of individual scores assigned to three different parameters: congestion (Suzuki et al. 1993; Ishikawa et al. 2025) (none = 0, slight = 1, mild = 2, moderate = 3, severe = 4), vacuolation (none = 0, slight = 1, mild = 2, moderate = 3, severe = 4), and necrosis (none = 0, single-cell necrosis = 1, less than 30% = 2, 30%-60% = 3, greater than 60% = 4). Scores for each finding ranged from 0 to 4, with a maximum score of 12. Each 100x liver field was scored, and the mean value was calculated from 10 microscopic fields.

### Terminal Deoxynucleotidyl transferase dUTP Nick end labeling assay

Terminal deoxynucleotidyl transferase dUTP nick end labeling (TUNEL) staining procedure was performed on 5-µm sections of liver tissues using a commercially available fluorescein In Situ Cell Death Detection Kit (Roche Diagnostics, Indianapolis, IN), following the manufacturer’s instructions. For the purpose of nuclear counterstaining, 4', 6-diamidino-2-phenylindole (DAPI) (Vectashield Antifade Mounting Media; H-2000) was utilised. TUNEL-positive cells were observed in 10 fields/section under a fluorescence microscope (magnification 200x) and quantified using ImageJ, Fiji software (version 2.1.051) (Ishikawa et al. 2025).

### Statistical analysis

Statistical analysis was performed using Prism 10 (GraphPad Software, LLC, San Diego, CA), ‘and p < 0.05 was considered statistically significant. The Shapiro-Wilk test was performed to check normality. The values for the mean and the standard error of the mean are presented. ANOVA was used for one-way comparison among multiple groups, and the significance was determined by the Tukey method. The Student’s t-test was applied for two-group comparisons. All experiments were repeated at least two times.

## Results

### Neutrophils stimulated by inflammatory IELs produce NETs and migrate to the liver

**Fig. 1:**
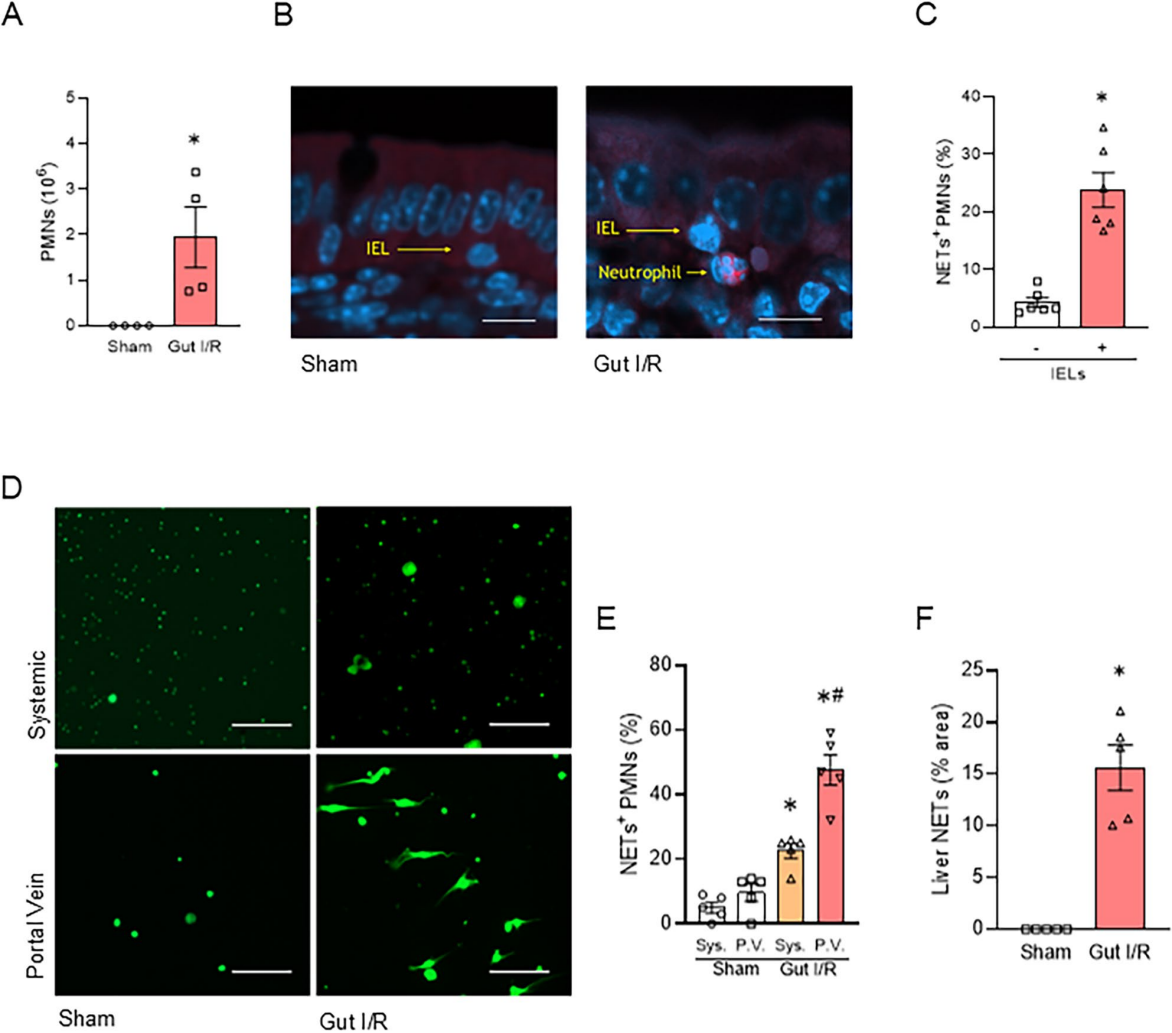
Neutrophils stimulated by inflammatory IELs produce NETs and migrate to the liver.** (A)** Flow cytometric quantification of neutrophils in the whole intestinal epithelial layer of sham and gut I/R mice (*n* = 4 per group). The Student’s t-test was applied. *p < .05 versus sham. **(B)** Representative immunofluorescence images of small intestinal sections stained for Ly6G (red) and DAPI (blue), showing the colocalization of neutrophils and IELs in the mucosal layer. Scale bar, 10 μm. **(C)** Quantification of NET⁺ neutrophils in systemic blood cultured alone or co-cultured with IELs. The Student’s t-test was applied. *p < .05 versus without IELs. **(D)** Representative fluorescence microscopy images of NETs formation in neutrophils in systemic (Sys.) and portal venous (P.V.) blood 4 h after reperfusion (SYTOX Green staining). Scale bar, 100 μm. **(E)** Quantification of NET⁺ neutrophils in systemic and portal venous blood 4 h after reperfusion, determined by flow cytometry (*n* = 5 per group). ANOVA was used for one-way comparison among multiple groups, and the significance was determined by the Tukey method. *p < .05 versus respective shams, ^#^*p* < .05 versus P.V. of gut I/R. **(F)** Quantification of NETs area in the liver. NETs in the liver of gut I/R were significantly increased compared to sham. The Student’s t-test was applied. *p < .05 versus sham

We first examined the interaction between neutrophils and IELs in the gut during gut I/R. Flow cytometric analysis revealed that the number of neutrophils significantly increased in the whole intestinal epithelial layer in gut I/R compared with sham (Fig. 1A). To confirm their localization, immunostaining of the small intestinal tissues was performed. It was shown that neutrophils co-localized with IELs within the intestinal mucosa, suggesting a close interaction between the two cell populations (Fig. 1B). Next, we co-cultured neutrophils and IELs isolated from gut I/R mice in vitro. Neutrophils stimulated by IELs produced a significantly greater amount of NETs (Supplementary Fig. 1A, Fig. 1C). Then, we examined NET-positive neutrophils in the systemic circulation and the portal circulation, which carries gut-derived blood. NET-positive neutrophils were significantly more abundant in the portal circulation than in systemic circulation, suggesting translocation of activated neutrophils from the intestine to the liver (Fig. 1D-E). Additionally, immunohistochemical staining demonstrated an increased NETs in the liver of gut I/R mice (Fig. 1F, Supplementary Fig. 1B). Taken together, these findings indicate that acute inflammation promotes close interactions between neutrophils and IELs in the intestinal mucosa, leading to the formation of NETs and the release of NET-expressing neutrophils into the portal circulation, and subsequently into the liver.

### IEL-primed neutrophils exacerbate liver inflammation

**Fig. 2:**
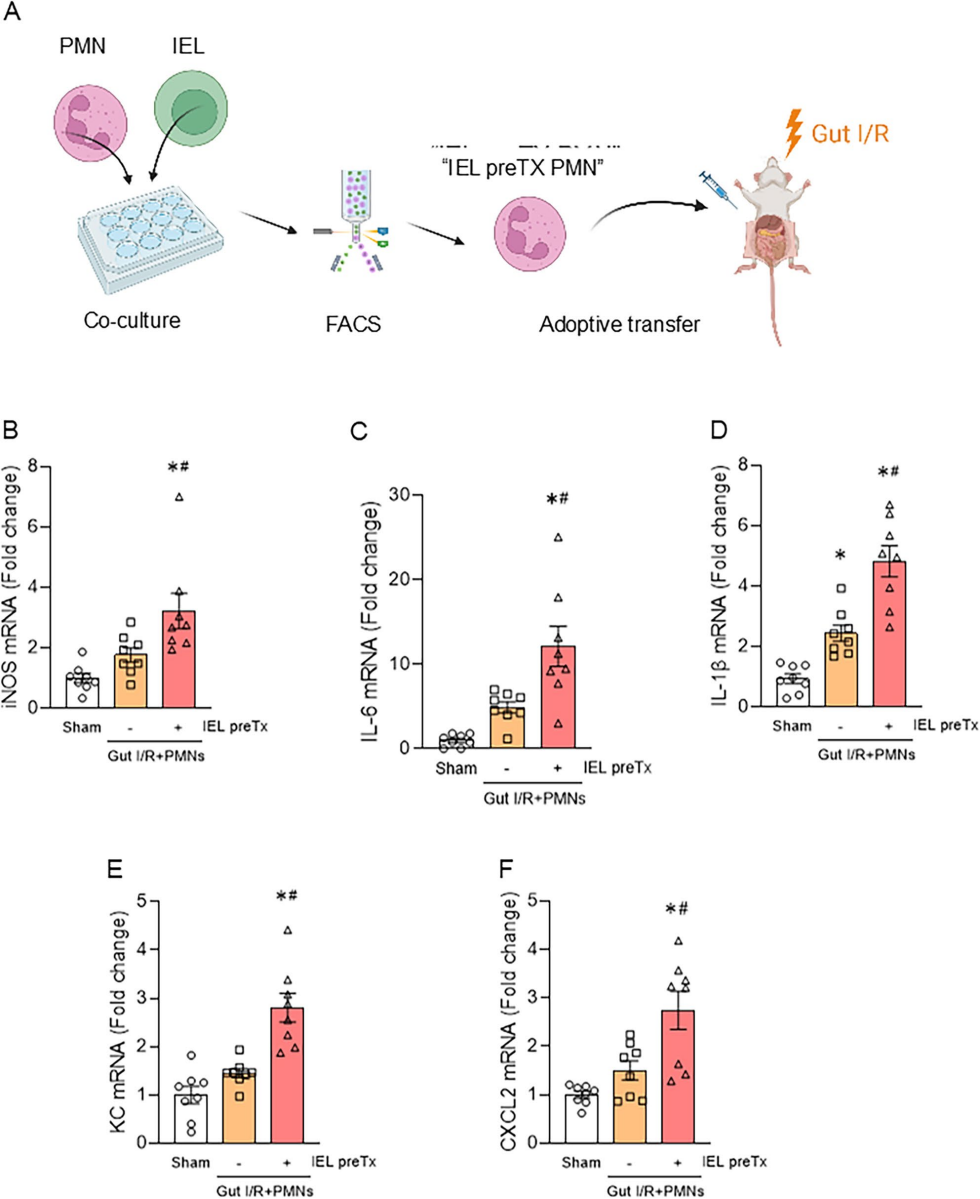
IEL-primed neutrophils exacerbate liver inflammation.** (A)** Schematic diagram of the adoptive transfer model. Neutrophils were co-cultured with or without IELs, sorted by FACS, and intraperitoneally injected into gut I/R mice. **(B–F)** Hepatic mRNA expression levels of iNOS **(B)**, IL-6 **(C)**, IL-1β **(D)**, KC **(E)**, and CXCL2 **(F)** 20 h after reperfusion, measured by qPCR and normalized to β-actin (*n* = 8 per group). ANOVA was used for one-way comparison among multiple groups, and the significance was determined by the Tukey method. *p < .05 versus sham, ^#^*p* < .05 versus without IEL preTx; iNOS, inducible NO synthase; IL, interleukin; KC, keratinocyte-derived chemokine; CXCL2, C-X-C motif chemokine ligand 2; mRNA, messenger RNA

Next, we explored the impact of the NETotic neutrophils induced by IELs, i.e., “IEL-primed neutrophils,” on liver injury using an in vivo adoptive transfer model. IELs and neutrophils were first co-cultured in vitro to generate IEL-primed neutrophils, which were subsequently labeled with Ly6G, a neutrophil marker, and separated by cell sorting. These IEL-primed neutrophils were then adoptively transferred into mice subjected to gut I/R injury **(**Fig. 2A**)**. In mice receiving IEL-primed neutrophils, hepatic expression of iNOS mRNA, a marker of M1 macrophage polarization, was significantly higher than in mice receiving unstimulated neutrophils **(**Fig. 2B**)**. Moreover, the expression levels of proinflammatory cytokines IL-6 and IL-1β, as well as chemokines KC and CXCL2, were markedly elevated **(**Fig. 2C–F, Supplementary Fig. 3). These findings indicate that neutrophils primed by IELs exacerbate hepatic inflammation following intestinal injury.

### Discovery of the novel CD112-derived peptide DPX2 which attenuates IEL-induced NETs

**Fig. 3:**
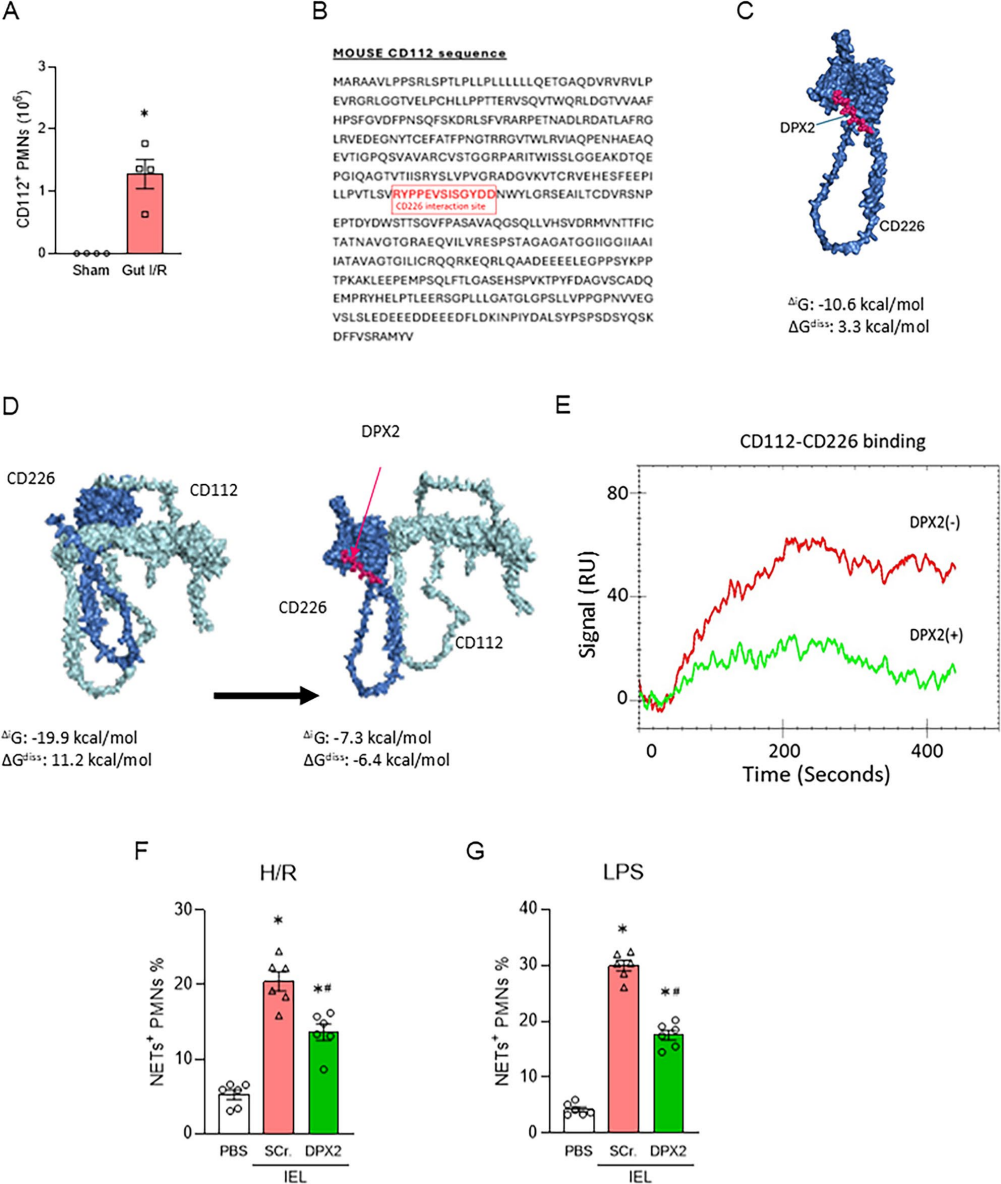
CD112-derived peptide DPX2 disrupts CD112–CD226 interaction and attenuates IEL-induced NETs.** (A)** Flow cytometric analysis of CD112-expressing neutrophils isolated from the whole intestinal epithelium of sham and gut I/R mice (*n* = 4 per group). The Student’s t-test was applied. *p < .05 versus sham. **(B)** The CD112 sequence, with highlighting residues 249–261 of CD112, was used to design DPX2 (RYPPEVSISGYDD). **(C)** Docking model and surface interaction view showing predicted binding of DPX2 to CD226. **(D)** Computing models of interaction between CD112 and CD226 with/without DPX2. **(E)** Surface plasmon resonance (SPR) analysis demonstrating that DPX2 competitively inhibits CD112–CD226 binding. Representative sensorgrams are shown. **(F**,** G)** Neutrophils were cultured with IELs with PBS, a scrambled peptide (Scr.), or DPX2 under hypoxia/reoxygenation **(F)** or in the presence of LPS **(G)** (*n* = 6 per group). ANOVA was used for one-way comparison among multiple groups, and the significance was determined by the Tukey method. *p < .05 versus PBS, ^#^*p* < .05 versus Scr. H/R, hypoxia/reoxygenation

We next sought to therapeutically target the IEL-primed neutrophils in gut I/R. We focused on neutrophil CD112, which is the molecule mediating the neutrophil-IEL interaction (Murao et al. 2025). We first confirmed that the population of CD112-expressing neutrophils was significantly increased in the whole gut epithelium of gut I/R model **(**Fig. 3A**)**. We then sought to inhibit the interaction between CD112 on neutrophils and its proinflammatory ligand CD226 expressed on IELs (Murata et al. 2023; Zhu et al. 2016). Computational modeling was employed to identify the binding interface between CD112 and CD226, from which we designed a CD112-derived peptide, termed DPX2 (amino acid sequence 249–261: RYPPEVSISGYDD) **(**Fig. 3B**)**. Computational modeling predicted that DPX2 strongly binds to CD226 and inhibits the interaction between CD112 and CD226 **(**Fig. 3C-D**)**. More importantly, surface plasmon resonance (SPR) analysis demonstrated that DPX2 competitively inhibits CD112–CD226 binding **(**Fig. 3E**)**. In vitro, DPX2 treatment markedly suppressed NET formation by neutrophils co-cultured with IEL under inflammatory conditions compared to the control groups **(**Fig. 3F, G, Supplementary Fig. 2). These results indicate that DPX2 effectively disrupts CD112–CD226 signaling and attenuates IEL-induced NET production.

#### DPX2 attenuates gut-derived NETs and liver inflammation

**Fig. 4:**
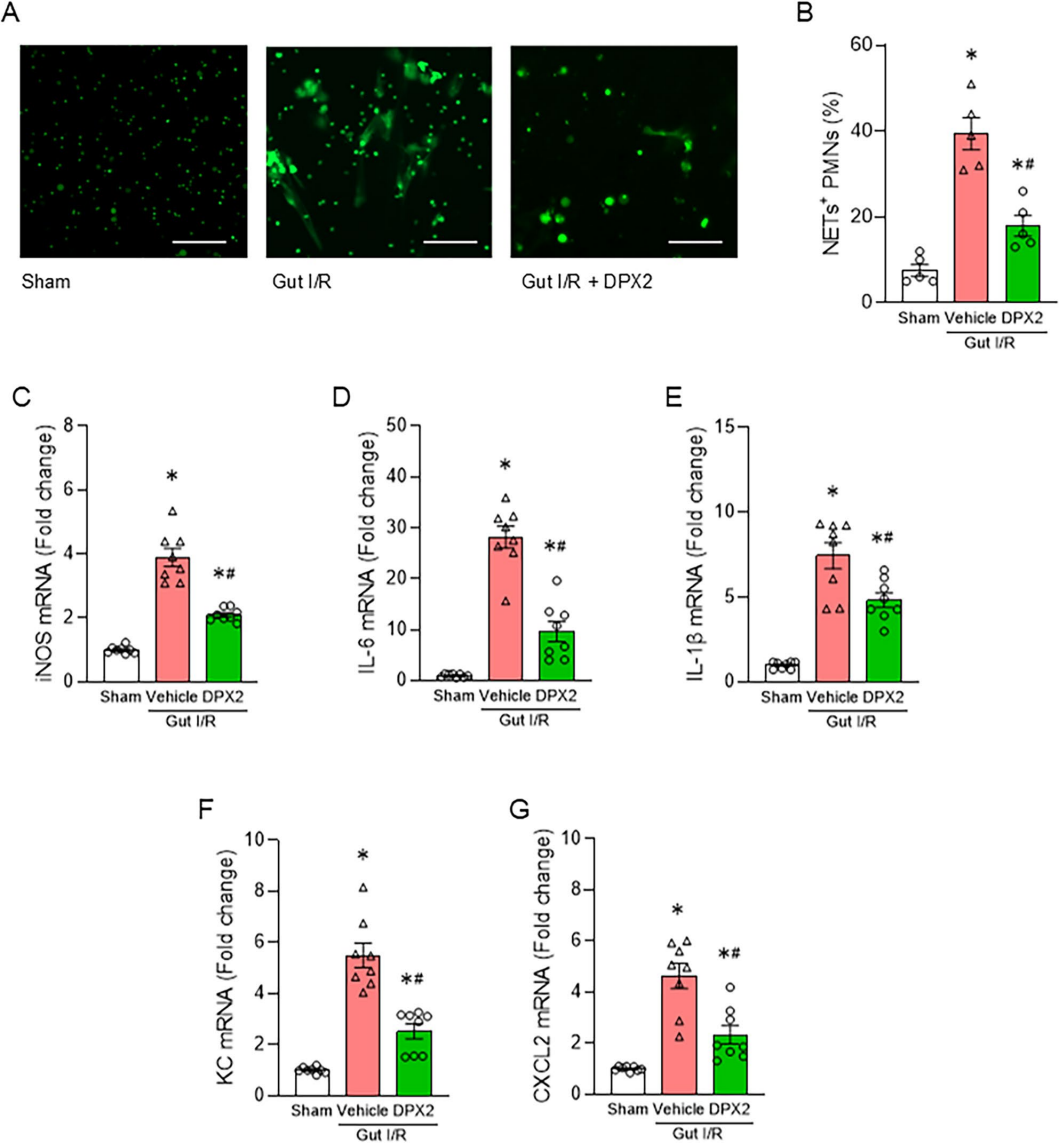
DPX2 attenuates gut-derived NETs and hepatic inflammation in vivo. **(A)** Representative fluorescence microscopy images of NETs formation in neutrophils in portal venous blood 4 h after reperfusion (SYTOX Green staining). Scale bar, 100 μm. **(B)** Quantification of NET⁺ neutrophils in portal blood from sham, gut I/R + vehicle, and gut I/R + DPX2 groups (*n* = 5 per group). **(C–G)** Hepatic mRNA levels of iNOS (C), IL-6 (D), IL-1β (E), KC (F), and CXCL2 (G) measured by qPCR 4 h after reperfusion (*n* = 8 per group). ANOVA was used for one-way comparison among multiple groups, and the significance was determined by the Tukey method. *p < .05 versus sham, ^#^*p* < .05 versus vehicle

We then performed in vivo experiments employing DPX2. We confirmed that DPX2 has no apparent toxicity as assessed by serum AST and ALT levels (Supplementary Fig. 4B, C). In the gut I/R model, administration of DPX2 immediately after reperfusion significantly reduced NETs detected in the portal circulation, indicating suppression of gut-derived neutrophil activation (Fig. 4A-B). Moreover, hepatic expression levels of iNOS, IL-6, IL-1β, KC, and CXCL2 were markedly decreased in DPX2-treated mice compared with the gut I/R control group, demonstrating that DPX2 alleviated liver inflammation mediated by gut-derived neutrophils (Fig. 4C–G, Supplementary Fig. 6). These data reveal that DPX2 effectively attenuates gut-derived NETs and liver inflammation.

### DPX2 mitigates liver injury following gut inflammation

**Fig. 5:**
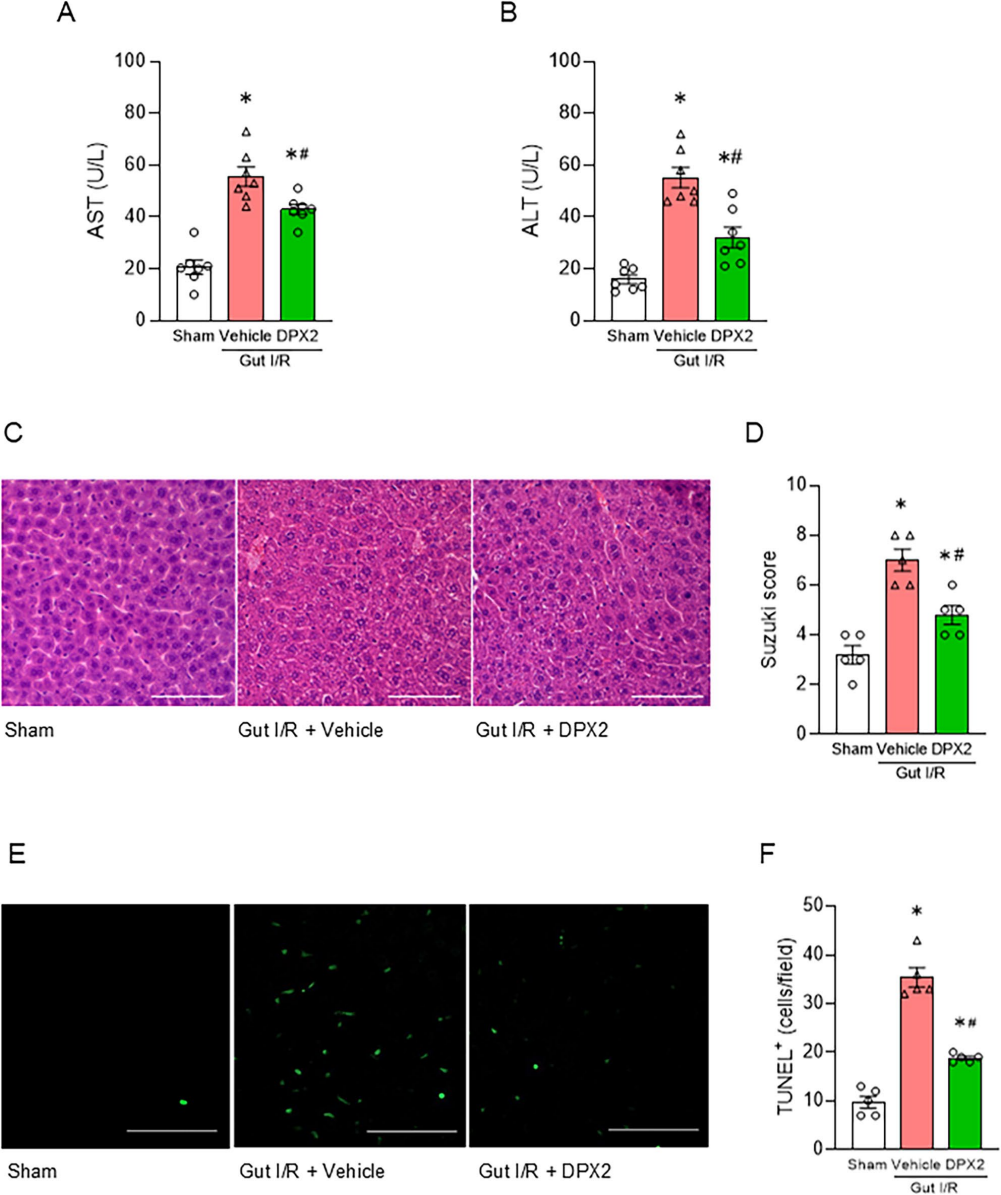
DPX2 mitigates liver injury following gut inflammation.** (A**,** B)** Plasma levels of AST **(A)** and ALT **(B)** in sham, gut I/R + vehicle, and gut I/R + DPX2 mice (*n* = 7 per group). ANOVA was used for one-way comparison among multiple groups, and the significance was determined by the Tukey method. *p < .05 versus sham, ^#^*p* < .05 versus vehicle. **(C)** Representative H&E-stained liver sections showing reduced hepatocellular necrosis and vacuolation in DPX2-treated mice. Scale bar, 100 μm. **(D)** Quantification of histological liver injury using the Suzuki score. ANOVA was used for one-way comparison among multiple groups, and the significance was determined by the Tukey method. *p < .05 versus sham, ^#^*p* < .05 versus vehicle. **(E)** Representative TUNEL staining of liver tissue sections indicating decreased hepatocyte apoptosis with DPX2 treatment. Scale bar, 100 μm. **(F)** Quantification of TUNEL⁺ cells per high-power field (HPF). TUNEL staining demonstrated a significant diminution of TUNEL-positive cells in the DPX2-treated group, confirming the suppression of hepatocyte apoptosis. ANOVA was used for one-way comparison among multiple groups, and the significance was determined by the Tukey method. *p < .05 versus sham, ^#^*p* < .05 versus vehicle

We further investigated the effect of DPX2 on liver injury. DPX2 treatment reduced plasma levels of the liver injury markers AST and ALT in gut I/R mice, indicating attenuation of hepatocellular damage **(**Fig. 5A-B**)**. Histological examination with H&E staining revealed that vacuolization and necrosis observed in the vehicle group were alleviated in DPX2-treated mice, accompanied by a significant reduction in the Suzuki score **(**Fig. 5C-D**)**. Furthermore, TUNEL staining showed a marked decrease in TUNEL-positive cells in the DPX2-treated group, confirming suppression of hepatocyte apoptosis **(**Fig. 5E-F**).** These findings demonstrate that　DPX2 ameliorates liver injury following gut inflammation.

## Discussion

**Fig. 6:**
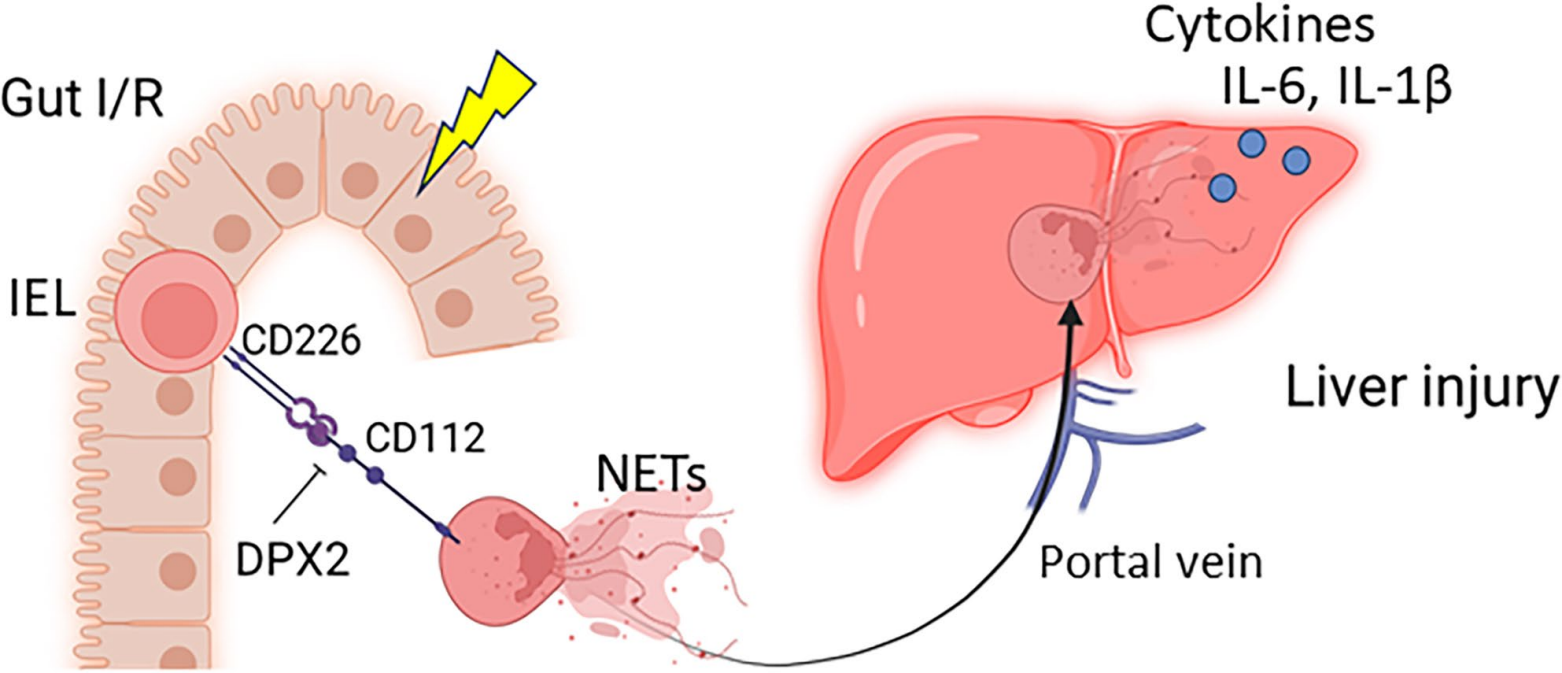
Targeting gut IEL-primed neutrophils to attenuate proinflammatory gut-liver crosstalk. In the course of intestinal inflammation, neutrophils interact with IELs via CD112, leading to enhanced NETosis. Neutrophils that undergo NET formation migrate from the intestine to the liver, contributing to secondary liver injury. The CD112-derived peptide DPX2 inhibits neutrophil priming by IELs and attenuates the proinflammatory gut-liver crosstalk

In this study, we demonstrated that neutrophils primed by IELs contribute to further liver injury during gut I/R. Microscopic analysis revealed colocalization of neutrophils with IELs within the intestinal mucosa. IEL-primed neutrophils, which were shown to form NETs at higher levels, were enriched in the portal circulation and accumulated in the liver. Adoptive transfer of IEL-primed neutrophils amplified hepatic expression of inflammation-related genes, including the M1 polarization marker iNOS, proinflammatory cytokines, and chemokines. Importantly, targeting the CD112–CD226 interaction with the novel CD112-derived peptide, DPX2, abrogated IEL-induced NETosis in vitro, reduced portal vein NETs, attenuated liver inflammatory and injury gene expressions, and transaminase levels, and improved histological features of liver injury. These findings reveal the previously underappreciated role of neutrophils in gut–liver crosstalk, illuminating a mechanistic axis through which local intestinal immune–neutrophil interactions propagate liver injury, and identify CD112-mediated signaling as a promising therapeutic target to mitigate secondary liver damage **(**Fig. 6**).**

Neutrophils are highly responsive to inflammatory mediators and rapidly migrate to sites of inflammation (Kolaczkowska and Kubes 2013; Oliveira et al. 2016). Our findings confirmed a novel mechanism in which neutrophils that infiltrate the intestinal tissue during inflammation can become primed through interactions with IELs, particularly via CD112–CD226 signaling. These primed neutrophils, which possess a NET-forming phenotype, subsequently re-enter the circulation through the portal venous system. This has been demonstrated by the observation of increased NET-positive neutrophils in the portal circulation compared to the systemic circulation, and the subsequent accumulation of NETs within liver tissue. This finding is consistent with the established observation that neutrophils that have emigrated from blood vessels can, in some cases, re‑enter the vascular compartment (Woodfin et al. 2011; Hirano et al. 2016), a concept known as neutrophil reverse transendothelial migration.

NETs formation primarily serves as a defense mechanism against pathogens (Papayannopoulos 2018); however, our results suggest that excessive NETs production induced by interactions with IELs can become pathologic. Moreover, even if NETs capture pathogens, NETs may carry the pathogens and drift through the circulation. While neutrophils were traditionally thought to die after releasing NETs (Remijsen et al. 2011), recent studies have shown that some neutrophils do not die immediately after NETs release, maintaining their mobility and expanding NETs coverage to prevent bacterial spread. Our findings are consistent with this new theory since we observed NETotic neutrophils migrating in the portal vein. Furthermore, in our previous studies on sepsis, we observed that NETs activate Kupffer cells via PAR-1, thereby promoting M1 polarization and inducing cytokine production (Murao et al. 2025) These reveal a previously unrecognized mechanism of inflammatory amplification within the gut-liver axis. The primary focus of this paper was gut-liver crosstalk, but IEL-induced NETs may contribute to pathophysiology in different organs as well. Some NETotic neutrophils may remain in the gut to further exacerbate the local injury. Gut-derived neutrophils in the liver may further migrate out from the liver to cause tissue injury in other distant organs, such as the lungs and kidneys, which could also be affected during systemic inflammation, including gut I/R injury.

The clinical significance of this study is that the CD112-derived peptide DPX2 effectively attenuates secondary liver injury driven by IEL-activated neutrophils. Although systemic corticosteroids are commonly used to blunt widespread inflammation and protect multiple organs (Cain and Cidlowski 2017; Annane et al. 2018; Venkatesh et al. 2018), their broad inhibition of cytokine pathways and suppression of neutrophil function can increase susceptibility to infection (Leung et al. 2025). Therefore, clinicians should exercise caution when considering systemic steroid therapy for preventing secondary liver damage. Conversely, selective blockade of the CD112–CD226 axis offers a targeted strategy, as it impedes the pathological activation of IEL-stimulated neutrophils while preserving essential neutrophil functions required for host defense. This more focused approach may reduce collateral immunosuppression and therefore holds considerable promise for translation into clinical practice.

While AST and ALT are clinically considered liver enzymes, they could also leak from the damaged intestinal smooth muscle cells/tissues. Therefore, we evaluated liver injury by a combination of serum liver enzymes and hepatic tissue inflammation markers and histology, including H&E and TUNEL staining. We acknowledge that a gut I/R model can create hepatic hemodynamic changes, which can be confounding factors. However, even though ligation of the superior mesenteric artery (SMA) reduces superior mesenteric venous return, it only leads to a partial, rather than direct or complete, reduction in portal venous flow to the liver. This partial reduction is compensated by the hepatic arterial buffer response (HABR), which leverages the liver’s dual blood supply (Walrand et al. 2021). In our adoptive transfer model, we have clearly demonstrated that IEL-primed neutrophils significantly exacerbated liver inflammation compared to control neutrophils, even though both mice were subjected to the same gut I/R procedure and, therefore, experienced comparable ischemic and hemodynamic stress. These results indicate that immune crosstalk is indeed a critical factor, driving gut I/R-induced liver injury. We acknowledge that the adoptive transfer approach represents an artificial experimental setting. Comprehensive profiling, including hemodynamic evaluation, would be particularly valuable in further strengthening our findings.

It is well-known that gut ischemia-reperfusion can cause significant changes in the composition and function of the gut microbiota, known as dysbiosis (Hu et al. 2022; Dai et al. 2022). Dysbiosis can potentially contribute directly to systemic inflammation and the propagation of inflammation to the liver by disrupting gut barrier function and facilitating the translocation of endotoxins (LPS) and other microbial metabolites into the bloodstream (Hsu and Schnabl 2023; Vincenzo et al. 2024; Bousbaine et al. 2022). Although this study did not assess the gut microbiota, it showed that neutrophils activated via CD112 undergo NETosis and migrate to the liver through the portal vein, suggesting that DPX2 reduces secondary liver injury independently of the microbiota. While interventions targeting the gut microbiota might involve treatments administered via the gastrointestinal tract, such methods can be challenging when hemodynamics are unstable (Reintam Blaser et al. 2017). Therefore, proposing a therapeutic intervention targeting the priming of neutrophils and IELs, independent of the microbiota, could represent a significant step forward for future treatments.

In our previous research on gut-liver crosstalk in sepsis, neutrophils were characterized as the mediators of injury, while Kupffer cells were viewed as the responders to neutrophil-induced stimuli and the subsequent cause of liver inflammation (Murao et al. 2025). Consistent with this prior study, we found neutrophils as the mediators of the crosstalk mechanism in this study, but this does not exclude the involvement of Kupffer cells in gut I/R-induced liver injury. Rather, based on our earlier findings, it is reasonable to consider Kupffer cells as the primary cellular source of inflammation in the liver following neutrophil activation. In the context of gut I/R injury, neutrophils are well-documented for their rapid recruitment to the intestinal mucosa via endothelial activation and complement/cytokine production, subsequently infiltrating both local and distant organs (Wang et al. 2024). As pivotal innate immune cells and often initial responders contributing to tissue damage (Fine et al. 2020), their established role underpins our focus on neutrophils in the present study. While other cell types, such as monocytes, likely contribute to gut I/R-induced liver injury, our specific objective is to elucidate the role of neutrophils in this pathology. To address potential bias in our targeted approach, a more comprehensive profiling would be beneficial to confirm the dominance of this neutrophil-driven pathway in the gut I/R model. Future studies on other cell types will undoubtedly deepen our understanding of this intricate mechanism.

Several limitations should be considered. We acknowledge the lack of an unbiased screening process, including multi-omic analyses across various cell types. However, our targeted approach is a direct and logical extension of our recent work in sepsis. The primary objectives of the current study were to mechanistically investigate liver injury focusing on gut-primed neutrophils using a sterile gut I/R model and to transition from mechanism discovery to investigating a therapeutic intervention—namely, the protective effects of DPX2—focusing on the specific axis involving neutrophils and IELs previously identified. In parallel, akin to gut I/R model, in a pilot study using experimental sepsis model, we also confirmed the beneficial outcomes of DPX2, indicating its therapeutic potential in acute inflammatory and infectious disease conditions (unpublished data). Next, the pharmacokinetics of DPX2 require further investigation. While the intraperitoneal administration of 1.0 µg/g has proven effective, there is a need to investigate dose-response benefits to determine its optimal dosage. Additionally, while evaluating the long-term therapeutic effects of DPX2 and its impact on chronic inflammation might be necessary, it is also notable that secondary liver injury significantly influences acute-phase survival rates, and current treatments for liver injury remain supplementary. Thus, there is considerable anticipation for new drugs targeting novel mechanisms. Furthermore, this study relies entirely on animal models, which may not fully replicate human pathophysiology. While animal studies are essential, there can be significant differences in cellular mechanisms between species that could impact the applicability of findings to human therapy. However, CD112 is expressed not only in mice but also in humans. Therefore, it is certainly worthwhile to investigate the present mechanism and therapeutic potential in humans, including human tissue culture and organ-on-a-chip models.

In conclusion, neutrophils primed by IELs in the injured gut form NETs and migrate to the liver via the portal circulation, driving secondary liver inflammation and injury. Targeting CD112–CD226 interactions with the CD112-derived peptide DPX2 suppresses the NET formation, reduces portal and hepatic NETs, and ameliorates liver inflammation and damage. These findings delineate a novel cellular mechanism of gut–liver crosstalk and identify CD112 signaling as a promising target to prevent gut-derived liver injury.

## Supplementary Information

Supplementary Material 1.

## Data Availability

No datasets were generated or analysed during the current study.

## Abbreviations

NETs: neutrophil extracellular traps
IEL: intraepithelial lymphocyte
PMN: polymorphonuclear neutrophil
I/R: ischemia-reperfusion
Sys: systemic
P.V.: portal vein
SPR: surface plasmon resonance
AST: aspartate aminotransferase
ALT: alanine aminotransferase
Cit-H3: citrullinated histone H3
