# Supplementary material for: A novel CD112-derived peptide targeting gut-primed neutrophils to attenuate deadly hepatic injury
Source: Mol Med. 2026 Feb 17;32:25. doi: 10.1186/s10020-026-01426-3 (PMC12914937; doi:10.1186/s10020-026-01426-3)
Supplement: Supplementary file 1 — Supplementary Material 1. [file 10020_2026_1426_MOESM1_ESM.pdf]

Supplementary Figure 1

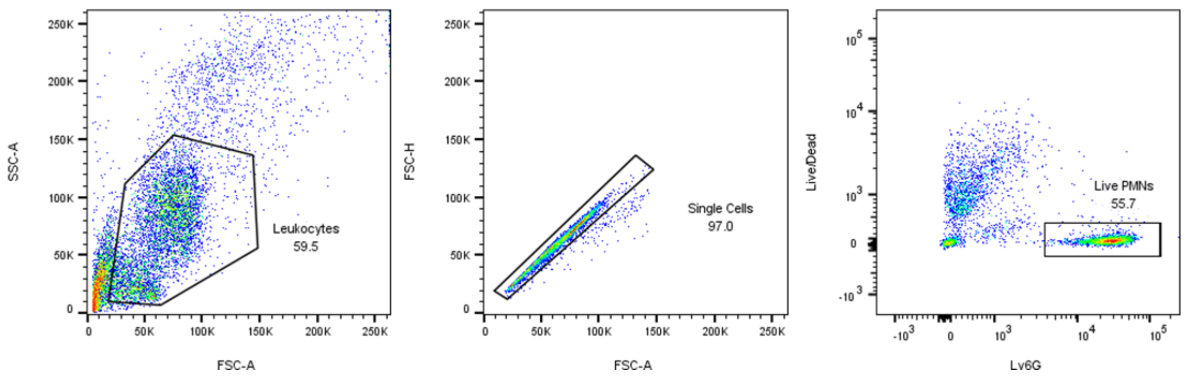

Supplementary Figure 1. The representative gating strategy of flow cytometry plots for identifying live neutrophils.

Supplementary Figure 2

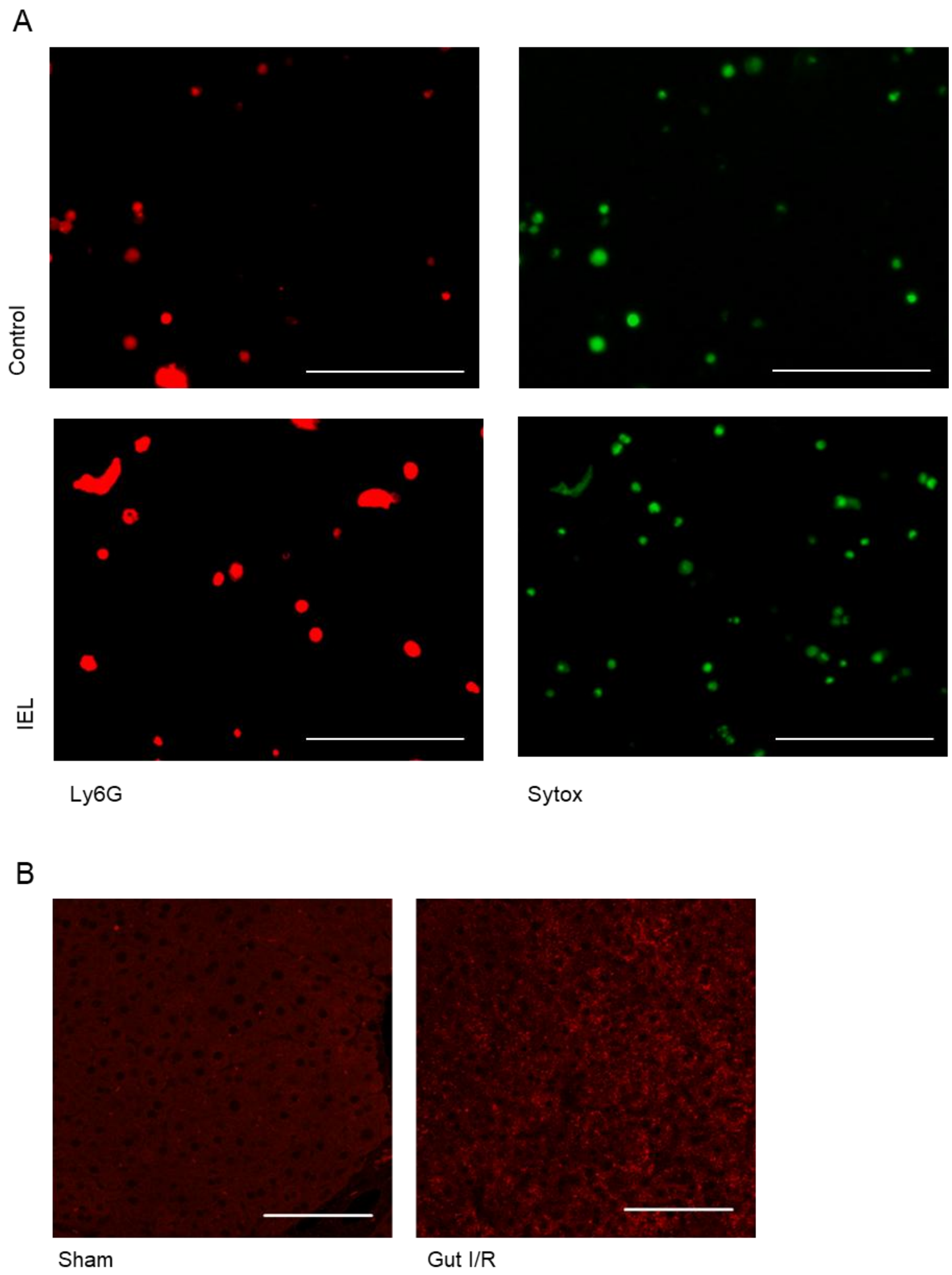

Supplementary Figure 2. (A) Representative fluorescence microscopy images of NETs formation in neutrophils co-cultured with IELs isolated from gut I/R mice. Scale bar, 200 μm.

(B) Representative fluorescence microscopy images of NETs formation in the liver tissue of gut I/R mice (citrullinated histone H3 staining; Cat. No.: ab5103, Abcam, Cambridge, UK). Scale bar, 100 μm; NETs, neutrophil extracellular traps; IEL, intraepithelial lymphocytes; I/R, ischemic reperfusion.

.

Supplementary Figure 3

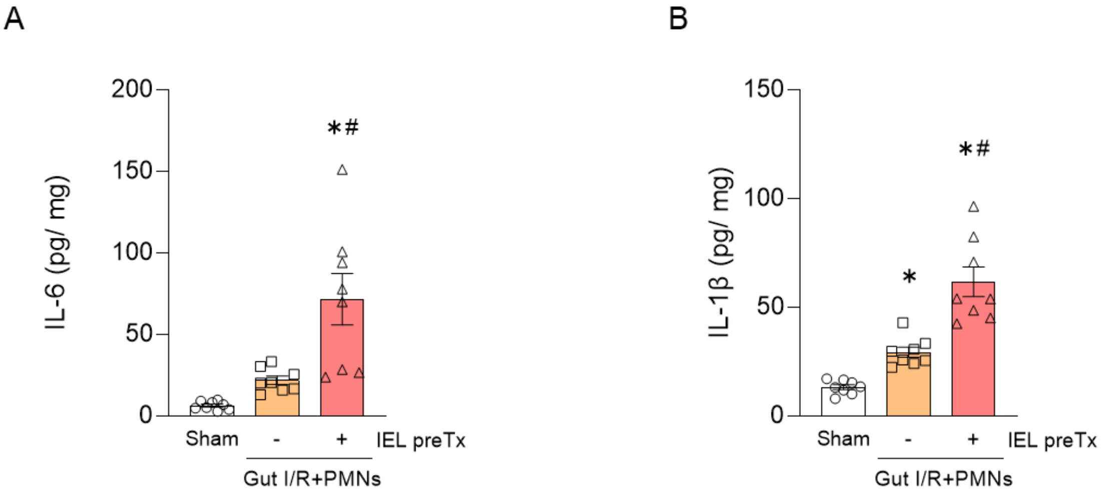

Supplementary Figure 3. Hepatic protein levels of IL-6 **(A)** and IL-1β **(B)** in gut I/R mice injected with neutrophils (n = 8 per group) measured by mouse IL-6 ELISA kit (Cat. No.: 555240; BD Biosciences) and IL-1β ELISA kit (Cat. No.: 432616, Biolegend). ANOVA was used for one-way comparison among multiple groups, and the significance was determined by the Tukey method. *p < .05 versus sham, #p < .05 versus without IEL preTx; IL, interleukin; ELISA, enzyme-linked immunosorbent assay.

Supplementary Figure 4

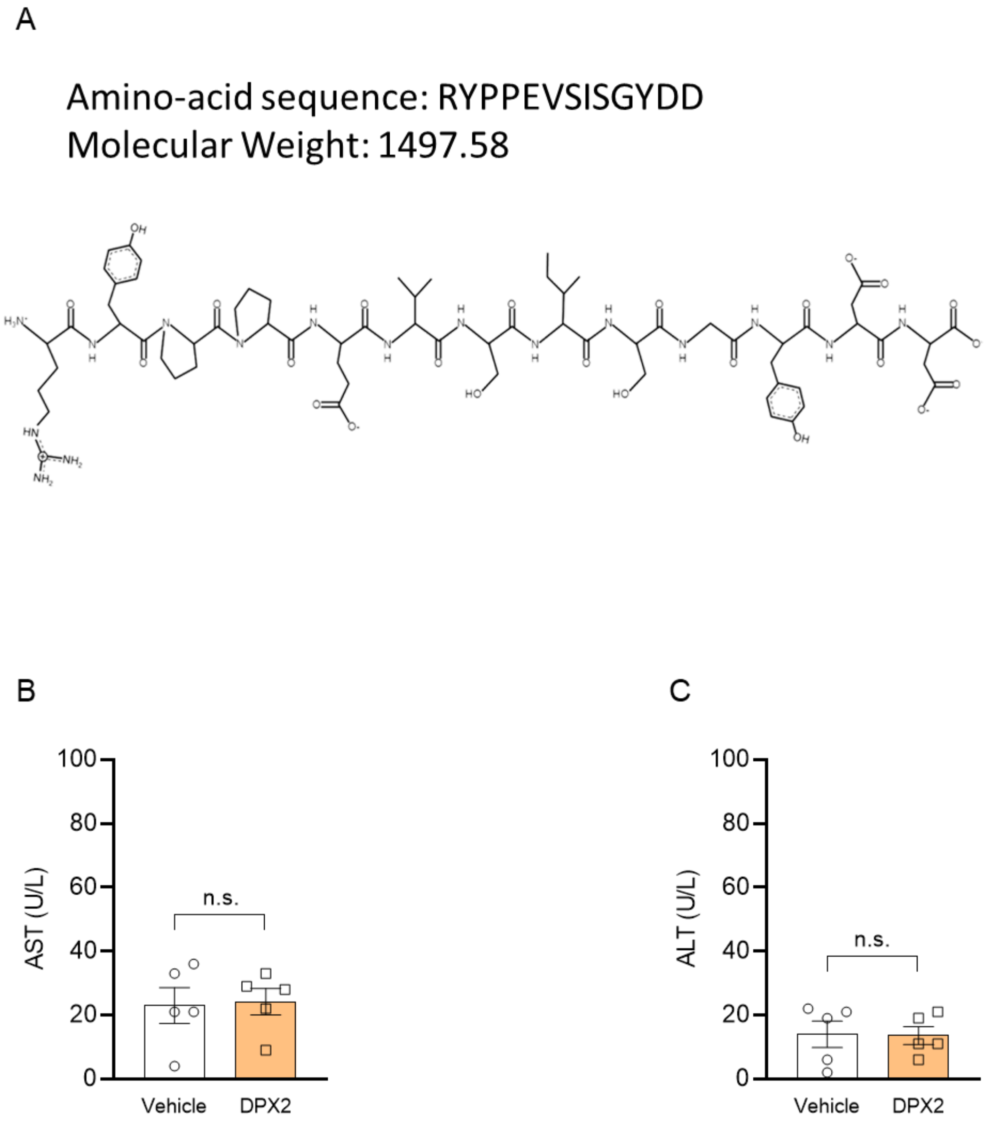

Supplementary Figure 4. (A) The full chemical structure, the amino-acid sequence, and molecular weight of DPX2. **(B, C)** Plasma levels of AST **(B)** and ALT **(C)** in sham mice receiving vehicle or DPX2 (n = 5 per group). The Student's t-test was applied; n.s. not significant; AST, aspartate aminotransferase; ALT, alanine aminotransferase.

Supplementary Figure 5

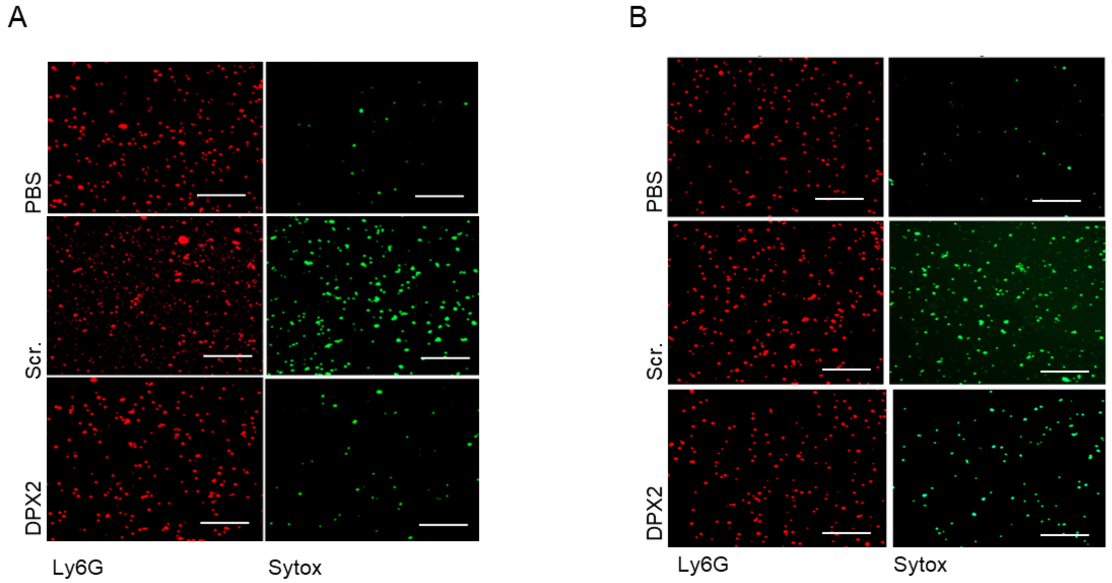

Supplementary Figure 5. Representative fluorescence microscopy images of NETs formation in neutrophils co-cultured with IEL in the presence of LPS **(A)** or under hypoxia-reoxygenation **(B)**. Scale bar, 100 μm.

Supplementary Figure 6

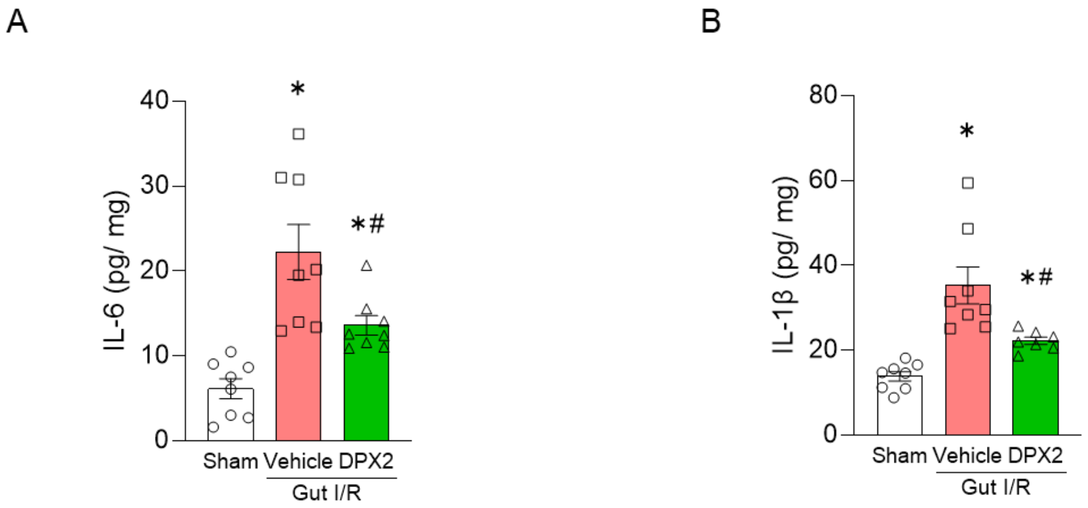

Supplementary Figure 6. Hepatic protein levels of IL-6 **(A)** and IL-1β **(B)** in gut I/R mice treated with DPX2 (n = 8 per group) measured by EISLA. ANOVA was used for one-way comparison among multiple groups, and the significance was determined by the Tukey method. *p < .05 versus sham, #p < .05 versus vehicle.
